# A Tribute to Peter H Seeburg (1944–2016): A Founding Father of Molecular Neurobiology

**DOI:** 10.3389/fnmol.2016.00133

**Published:** 2016-11-29

**Authors:** William Wisden

**Affiliations:** Department of Life Sciences and Centre for Neurotechnology, Imperial College LondonLondon, UK

**Keywords:** Peter Seeburg, obituary, glutamate receptors, GABA receptors, growth hormone, shotgun sequencing, RNA editing

*“Molecular approaches will continue by ingenious innovations to make inroads in neuroscience at the interface of physiology, cell biology and genetics. By its versatile nature, molecular biology ensures its contribution to our understanding of the workings of the brain. This is the good news! The bad news is that we need to wait to find out how.” (Seeburg, 2008)*.

On 22nd August 2016, the fields of molecular neurobiology and endocrinology lost one of their pioneers and true giants, Peter Seeburg, who died aged 72, a day after his birthday. His funeral ceremony took place in Heidelberg where he had worked since 1988, first as a professor at the University of Heidelberg (ZMBH) and then since 1996 as a director of the Max Plank Institute (Department of Molecular Neurobiology). Many of Peter's former colleagues, students and postdocs came together with his family members to celebrate his life. Touching eulogies were given by no less than two Nobel prize winners: the physiologist Bert Sakmann, who collaborated with Peter for many years, and the developmental biologist Christiane Nüsslein-Vollhard, who was a friend and fellow PhD student with Peter. His professional contemporary, Heinrich Betz, gave a warm and endearing assessment of Peter's contributions to the field of molecular neurobiology. One of Peter's sons, Daniel P. Seeburg, now a neuroradiologist in the USA, and biotechnologist Karoly Nikolics, one of Peter's friends from the days of Genentech, both emotionally summed up the warm and intense character of the man that many of his former students and postdocs knew.

## Seeburg career overview

Peter Seeburg pioneered the cloning and expression of recombinant hormones in *E. coli* for human use. Beforehand, doctors had had to use hormones and peptides purified from animals, sometimes with concurrent health risks for patients because of co-purified viruses or neurodegenerative agents. This recombinant expression work was transformative for biotechnology. In particular, in 1979, Peter developed the expression of recombinant growth hormone, which is still sold today (Goeddel et al., 1979). He and his colleagues made many contributions to the cloning of hormones and their receptors, and he was streets ahead of many of his contemporaries in applying cloning techniques to reveal the sequences of peptides, hormones, receptors and their gene structures. He also developed several seminal molecular biology methods, such as shotgun sequencing (Messing et al., 1981), later used in the genome sequencing programs. And so Peter, as one of the first biotechnologists and members of Genentech, was already well known in his early forties as a “molecular endocrinologist.” But perhaps we, as neuroscientists, know him best as one of the “godfathers” of molecular neurobiology. In the 1980s and 1990s, Peter was one of the first scientists to apply molecular biology techniques to characterize neurotransmitter receptors. His work, and that of the teams he assembled and inspired, brought us the molecular descriptions and functional characterization of nearly all the GABA_A_ receptor subunits, many of the ionotropic glutamate receptor subunits (AMPA, kainate, NMDA) and their properties, as well as the discovery and importance of RNA editing in the nervous system. Later he and his collaborators manipulated the expression and properties of these genes in mouse models, and extended the analysis of these subunits *in vivo*. His work set the foundations for much of the molecular biology of ligand-gated channels that is taken for granted as textbook knowledge today.

## Seeburg: career foundations in the Heinz Schaller lab

Peter Seeburg was born in August 1944 in Querfurt near Halle/Salle, in what became Eastern Germany (DDR) after World War II. At the end of the war his family came to the west and to Munich where he grew up. Between 1964 and 1967, he studied Chemistry at the University of Munich, and then from 1967 to 1972 studied Biochemistry at the University of Tübingen in the south west of Germany. He then joined the virologist Heinz Schaller's lab at the Max Planck Institute for Virus Research Tübingen as a PhD student. Peter's thesis work on viral promoters was published as several papers (e.g., Seeburg and Schaller, 1975). According to Nüsslein-Vollhard, the lab was exciting for many young biochemistry students. In the late 1960s, Schaller, who was one of the molecular biology pioneers, was giving lectures on DNA replication—an exotic topic back then—and that was probably why Peter joined the group. This was all before the modern methods of DNA sequencing and other molecular biology techniques were developed. On her Nobel laureate webpage Nüsslein-Vollhard wrote: “Although I was an experienced molecular biologist, I got bored with my projects at the end of my thesis (1973). The prospect of continuing the study of transcriptional control via the structure of promoter regions meant developing new methods for DNA sequencing. The field of recombinant DNA technology was growing and a fellow student and good friend, Peter Seeburg, argued strongly for it. I was skeptical, and at that early time, like most other people in Tübingen, did not foresee its powers.” But Peter did, and for him, that meant moving with a scholarship from the German Research Foundation (DFG) to Howard Goodman's lab in the Department of Biochemistry and Biophysics at the University of California, San Francisco (UCSF) where Peter continued to develop studies on cloning. At this time, Peter was the quintessential hippie, with rings and long hair, playing guitar, at one time in a band. With his celebrated sharp humor, he brought a consistent and characteristic intensity and practical brilliance to his lab work: smoking cigarettes, drinking coffee and pipetting simultaneously (with probably some other stimulants as well). His vigor, ambition and sense of humor were qualities that would inspire his future students and postdocs. In the Goodman lab, Peter was one of the first to use the new cloning techniques to get sequence information of hormones such as growth hormone (e.g., Seeburg et al., 1977).

## The genentech ERA: shotgun sequencing, growth hormone and neuroendocrinology

In 1978 Peter, then aged 34, joined the company Genentech in San Francisco. Genentech was a new breed of company, dedicated to turning the fruits of molecular biology into commerce. Together with colleagues such as his German contemporary Axel Ullrich, who pursued a parallel career to Peter with Goodman and then at Genentech (Ullrich et al., 1977; Sures et al., 1980), the late 1970s and 1980s were Peter's time to help pioneer biotechnology. During this period, Peter and colleagues developed several key molecular biology techniques, and one in particular, shotgun DNA sequencing, has been enduring (Messing et al., 1981). Rather than move linearly through a DNA sequence, building a new sequencing primer as each new part of the sequence was discovered, the shotgun method enabled rapid dideoxy-sequencing of randomly (hence “shotgunned”) cloned overlapping DNA fragments in piece-meal order, using just one sequencing primer. The computer could then be used to piece the multiple overlapping sequences together. Thus, sequences of cDNAs, such as those encoding the hormones that Peter's group were working on, could be efficiently assembled. The method was widely adopted. It was also the natural strategy for the genome sequencing efforts that came several decades later. Continuing his work from the Goodman lab (Seeburg et al., 1978), Peter's group was one of the first to express a recombinant protein in *E. coli*, a method which underpins the biotechnology industry today (Goeddel et al., 1979, 1980). This protein was growth hormone. This work is the foundation of Peter's early scientific reputation (Seeburg, 1986).

At Genentech, Peter cemented the high publication rate that would become a hallmark. For example, 1982 at Genentech was a typical “Seeburg year”: he helped provide the sequence of the Met- and Leu-enkephalin precursor genes (Comb et al., 1982); together with Richard Axel and colleagues he dissected the promoter elements of the growth hormone gene (Robins et al., 1982); he extended into the genetic causes of growth defects by looking at human families with growth hormone mutations (Phillips et al., 1982); and his group provided the entire (!) sequence of a viral genome, bovine papilloma virus type 1 (Chen et al., 1982). The underlying theme always being the application of cutting edge and high-throughput molecular biology techniques. He later developed an interest in Gonadotropin Releasing Hormone (GnRH), a peptide absolutely needed for reproduction. He worked on a spontaneous mutant mouse which did not have a functional GnRH gene, causing poorly developed gonads, and thus could not reproduce. Sensationally for the time, he and his collaborators could rescue the phenotype of this mouse by using a GnRH transgene placed into the genome of the mutant mouse (Mason et al., 1986). This transgene contained all the regulatory regions needed to express GnRH in the correct regions of the hypothalamus, and this expression restored the ability of the hypogonadal mouse to reproduce—in other words, this was one of the first successful attempts at gene therapy (Mason et al., 1986). He was to return to GnRH neurons over a decade later, when his group studied genetically labeled GnRH neurons in the hypothalamus using electrophysiology to profile the neurotransmitter receptors on these neurons to see how they secrete GnRH rhythmically (Spergel et al., 1999). Peter's last major Genentech study climaxed with the sequence of the luteal lutropin receptor, a large G-protein coupled receptor protein required for reproduction (McFarland et al., 1989).

## The molecular neuroscience ERA: “Seeburg never sleeps”

During his time at Genentech, through his work on hormones released from the hypothalamus and pituitary, Peter had become more widely interested in brain function. His interest was further stimulated when he was approached at Genentech by Eric Barnard, a British protein chemist and neurochemist. In the mid 1980s, little was known about molecular neuroscience. There were few sequences available of any large proteins expressed in the brain. Shosaku Numa and colleagues at the Department of Medical Chemistry at Kyoto University in Japan had published the sequences of the muscle nicotinic acetylcholine receptor and voltage-gated sodium channels and had set the standard high, having collaborated with Bert Sakmann on the electrophysiological properties of these recombinant receptors and ion channels (Sakmann et al., 1985; Mishina et al., 1986). Barnard, then based at Imperial College London, together with Ricardo Miledi, had pioneered injecting brain mRNA into *Xenopus* oocytes, which translated this into receptors that could be assayed electrophysiologically on the membrane of the cell (Sumikawa et al., 1981). Barnard's group were purifying the GABA_A_ receptor from bovine brains. In this they were successful (Sigel et al., 1983), but turned to Peter Seeburg for help with the cloning of receptor cDNAs. We need to remember that they asked Peter because at that time few had competence in this new area, molecular biology and cloning. Eric Barnard wanted to proceed quickly and effectively, since there was competition to clone the receptor from several other labs. Peter would have seemed the man to deliver—and he did. Peter Seeburg's team obtained sequences of peptide fragments from the affinity purified GABA_A_ receptor given to them by Barnard's group, which enabled Peter's team to make degenerate DNA primers, and screen cDNA libraries. The cDNA libraries required special care, because it was important that they contained full-length reading frames for large proteins, which was a rare thing at the time. The isolated GABA_A_ receptor α1 and β1 subunit mRNAs encoding the subunits were injected into *Xenopus* oocytes to assay their functional properties and pharmacology. The results became a full article in *Nature* detailing the sequence and some functional properties of the GABA_A_ receptor α and β subunits (Schofield et al., 1987). The article appeared back-to-back with one from Heinrich Betz's group giving the structure and sequence of the glycine receptor (Grenningloh et al., 1987), and it was apparent that together with the nicotinic acetylcholine receptor, these receptors formed a molecular superfamily. But this was only the start. In fact, it was the springboard for Peter's next career phase. In the late 1980s, Peter together with some talented colleagues, relocated to the ZMBH at the University of Heidelberg in Germany, where he set up a new lab (titled the Department of Molecular Neuroendocrinology) on the opposite side of the corridor from Heinrich Betz's group. The “endocrinology” part of the lab title was to prove largely historical.

As Betz pointed out in his eulogy, Peter Seeburg shook up the field of molecular neuroscience because of his intensity and organizational skills. Based on his experience and work ethic at an American biotech company, Genentech, he accelerated the pace at which sequences, subunits and their properties were determined. In particular, rather than assigning one postdoctoral RA or PhD student to do the whole project, or at least large amounts of it (as was perhaps the case in the Steve Heinemann or Heinrich Betz labs), Peter appointed a team of experts to take on different aspects of the project. Another unique feature of the time was organizing the lab with technical support, so that one technician ran the DNA sequencing, another looked after cell culture, etc. Having such a team, perhaps more commonplace now, was way ahead of the Zeitgeist then. “Seeburg never sleeps” was apparently a quote on the doors of some rival labs at the time. Each Seeburg paper was constructed as a team effort, passed on down the line: constructing cDNA libraries with full-length clones (the entire reading frame); screening the libraries with degenerate primers, subcloning the cDNAs into eukaryotic expression vectors, rapid DNA sequencing, transfection into mammalian cells with Genentech-based expression vectors for state-of-the-art patch-clamp electrophysiology, looking at the expression patterns of the mRNAs with *in situ* hybridization, site-directed mutagenesis to test functional properties of the subunits using ligand-binding or patch-clamp methods. The electrophysiology was a key part of the endeavor, and here of course, Bert Sakmann's input and collaboration was critical. The Sakmann lab was initially in Göttingen, and then relocated to Heidelberg—Sakmann says that he invited Peter to move to Göttingen, but Peter did not want to move, and so Sakmann moved to Heidelberg! In 1991, there was an amazing atmosphere when Sakmann and Erwin Neher received the Nobel prize for developing the patch-clamp technique, and there was a big party between the Sakmann and Seeburg labs.

## Seeburg lab life at ZMBH, at the university of Heidelberg

Seeburg lab life at ZMBH, at the university of Heidelberg. One of this manuscript's editors (JCM) told me that he was an undergraduate studying biology at that time at the University of Heidelberg, and remembers being inspired by the ideas and power Peter seeded and spread among the students at the ZMBH. The whole molecular neuroscience enterprise was driven by Peter's energetic nature. He did not like to be beaten and science was a race to be first. For the glutamate receptor field especially, Peter was worried. The labs of Steve Heinemann at the Salk Institute in La Jolla in California, and of the formidable molecular neurobiologist Shigetada Nakanishi at the Institute of Immunolgy at Kyoto University (Japan), who had previously competed with Peter on isolating peptide hormone cDNA sequences, were working on isolating neurotransmitter receptors. Nakanishi had invented expression cDNA cloning of receptors using the *Xenopus* oocyte system, and so could scoop Peter's team at any moment. In fact, Michael Hollmann and colleagues in Heinemann's group were first to the finish line, isolating by expression cloning, the first AMPA/kainate receptor subunit, GluA1, thus founding the subfield of glutamate receptor molecular biology (Hollmann et al., 1989). Nakanishi's group did indeed go on to identify the following year, also by expression cloning, the first NMDA receptor subunit GluN1 (Moriyoshi et al., 1991) and the first metabotropic glutamate receptor mGluR1 (Masu et al., 1991). So in 1990, given the competitive climate, Seeburg lab meetings for the people in his group working on glutamate receptors often took place at 9 a.m. Saturday mornings, which was a challenge because at that time in Germany, the shops were only open in the mornings on Saturdays. And on Sundays, Peter always arrived with cake for the lab at around 3 p.m. You were expected to be there. And if you were not, he would say on Monday, “I didn't see you yesterday, I thought you were interested in this project.” Many people benefited from Peter's fervor, including me, then a young and disorganized postdoc. A typical Seeburg weekday in the early 1990s, would be similarly intense. He would arrive in the lab at around 7 a.m. His first pleasure of the day was to open the X-ray film cassettes that were exposing the sequencing gels, develop the films, and read the gels over cigarettes and coffee into the computer, so that by the time the person leading on the project got into the lab, Peter was already ready to discuss the next stages of the work with them.

At ZMBH, Peter shared his office—whose door opened straight into the lab—with his chief postdoc and right-hand man Rolf Sprengel (who went onto share at least 92 publications with Peter). Peter ran an open door policy: anyone could go in with ideas, or to discuss data, at pretty much any time. Peter would often be observed from the vantage point of the lab through the open door standing at the phone, looking out the window smoking, and he was often heard talking to the editors of the *Nature* and *Science* journals. Alternatively, he might be seen reading or writing, or looking at X-ray films of sequencing gels on the lightbox, a cigarette always burning. He could also come out at any time into the lab, to ask detailed questions, to encourage, and to motivate. When the sequencing technician was on holiday, Peter would take over the lab sequencing himself, together with copious quantities of ^35^S, cigarette ash, coffee and acrylamide. Weekdays, Peter would leave around 7 p.m., after a 12-h day, sometimes collecting postdocs or students that were around and take them to dinner. Manuscripts were shaped up as the work progressed. Peter liked scientific writing and getting to the heart of a good sentence and its precise meaning. An enjoyable aspect of writing manuscripts with Peter was that phrases would be worked up through multiple drafts until the right level of precision was reached. He liked words and their meanings. His writing skill was consummate. With a word like “consummate,” for example, he would ponder it, underline it in red, light a cigarette, say it multiple times, blow out smoke, and say “yes, consummate, a good word.” Reading and languages remained a passionate hobby. In addition to his perfect midatlantic English, he enjoyed learning and speaking Russian, Italian and latterly he apparently took up and read some works in Latin. He was also fluent in the language of DNA, of all its codes and cryptic sequences. In 1990, Peter decided that Hannah Monyer and I needed to learn the genetic code off-by-heart. “GAA codes for what amino acid?” he would ask us; or, “Tell me what ‘CCC’ codes for?” Every day, one of these questions. We always got these wrong, which he found amusing and so decided that we were hopeless cases for passing the “DNA fluency” test. But one day I defeated him: “What does UGA mean?” I asked him. “Stop codon of course” he replied. “No!—it can be selenocysteine!” I quipped back with some satisfaction (I happened to have just read randomly in Nature that it was a newly discovered tRNA—Forchhammer et al., 1989), and so the master of molecular biology had to concede that he had learnt one (rare) codon from me!

**Figure 1:**
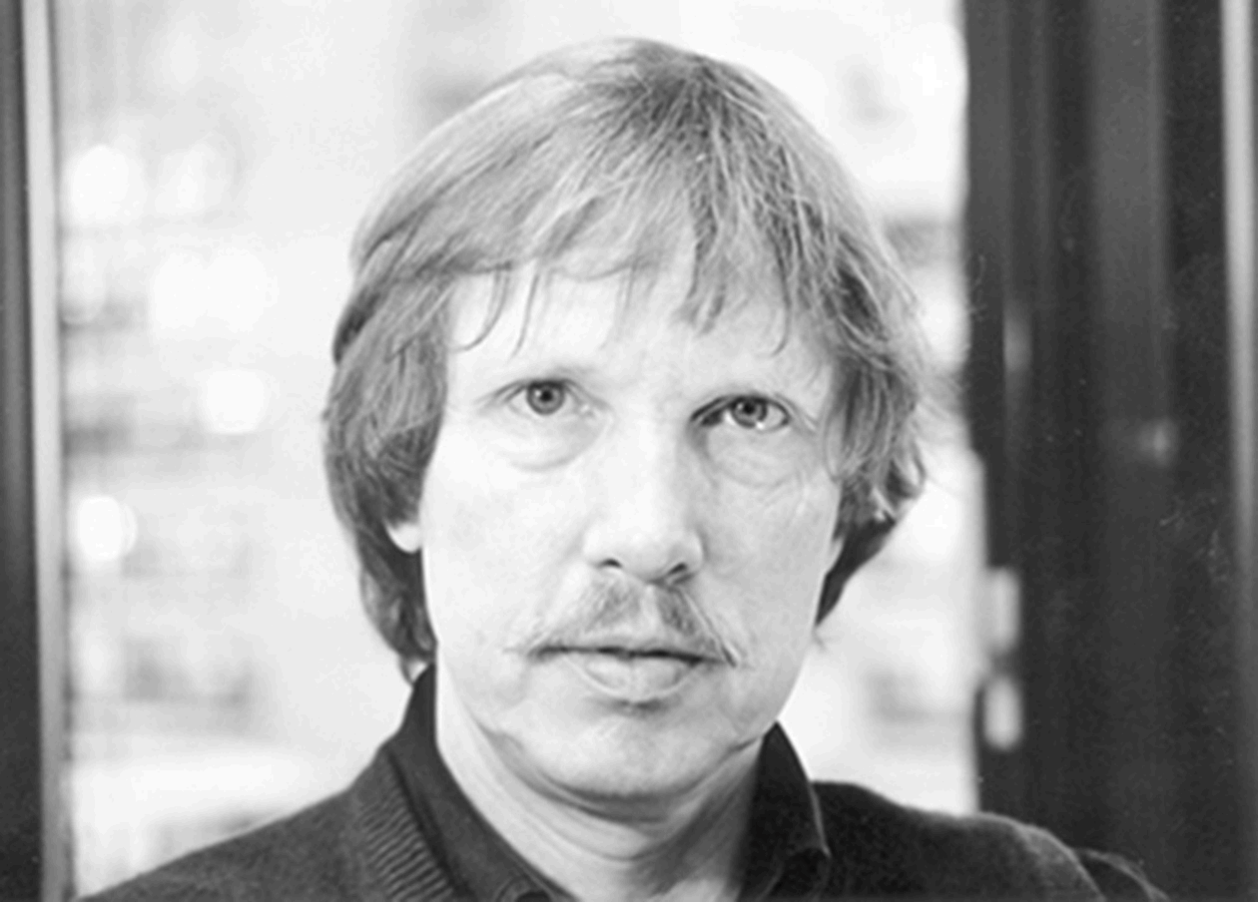
**Peter Seeburg in the ZMBH years, taken in 1993**. Reproduced with permission from Universitätsarchiv Heidelberg. Photograph: Michael Schwarz.

Peter's most prolific scientific period as a lab head in the field of molecular neurobiology was probably during the late 1980s to mid 1990s, when he was in his mid to late forties (see portrait in Figure 1). There were many key discoveries of this early molecular neurobiology period at ZMBH for Peter and his colleagues. It almost seemed like a *Nature, Science*, or *Neuron* paper per month, or sometimes several. The intensity of it was glorious. Some personal choices for GABA_A_ receptors are: cloning nearly the whole subunit family (e.g., Levitan et al., 1988; Pritchett et al., 1989a; Shivers et al., 1989; Seeburg et al., 1990); the use of mammalian cell lines (e.g., HEK293 cells) for expression of receptor subunits, now a routine method for all labs in this area (Pritchett et al., 1988); characterization of the benzodiazepine pharmacology of GABA_A_ receptors (Pritchett et al., 1989b; Lüddens et al., 1990; Korpi et al., 1993); identification of the R/H position in the GABA_A_ receptor α subunits that determined benzodiazepine sensitivity (Wieland et al., 1992), knowledge which allowed the group of Hanns Möhler in Zurich to do elegant work constructing a series of α subunit knock-in mice to test how benzodiazepines produce their selective effects such as sedation or anxiolysis (Rudolph et al., 1999; Löw et al., 2000); and mapping expression of GABA_A_ receptor gene family expression patterns (Wisden et al., 1992).

Some personal choices for Peter's impact on glutamate receptors are: AMPA, kainate and NMDA receptor gene heterogeneity (Keinänen et al., 1990; Werner et al., 1991; Herb et al., 1992; Monyer et al., 1992, 1994); the discovery of flip and flop splicing isoforms of AMPA receptor subunits and their functional significance (Sommer et al., 1990; Mosbacher et al., 1994); the discovery of selective Ca^2+^ permeability of some AMPA receptor subunit combinations (Verdoorn et al., 1991; Burnashev et al., 1992a); the probing of NMDA receptor channel structure to reveal the importance of a critical asparagine (Burnashev et al., 1992b; Kuner et al., 1996); the use of yeast two-hybrid screening to discover PSD95 binding to the receptor C-terminus of NMDA receptor subunits (Kornau et al., 1995); and as a little side line, in a short reinvigoration of his endocrinology days, the molecular description by expression cloning of the pituitary adenylyl cyclase-activating polypeptide (PACUP) receptor and its signaling (Spengler et al., 1993).

The crowning molecular biology glory, and in retrospect most novel finding of all, was the discovery of RNA editing and its molecular and enzymatic mechanisms, and this deserves some extra mention. Bernd Sommer in Peter's group discovered that a critical nucleotide, adenosine, is changed to inosine in the AMPA receptor GluR2 subunit nuclear transcripts by adenosine deaminase enzymes (Sommer et al., 1991; Higuchi et al., 1993; Melcher et al., 1996a). This A to I nucleotide transition, termed editing, effects a change in the codon for glutamine (Q) to arginine (R) in a critical transmembrane part of the GluR2 subunit and governs Ca^2+^ permeability (Burnashev et al., 1992a), and also influences receptor kinetics (Lomeli et al., 1994). Peter's group showed that nearly 100% of GluR2 transcripts are edited, and that when editing of GluR2 transcripts fails, which they arranged to happen in transgenic mice, it has a lethal effect because of the heightened Ca^2+^ entry into neurons (Brusa et al., 1995; Feldmeyer et al., 1999; Rosenthal and Seeburg, 2012). Even today, that 100% editing of the GluR2 gene takes place seems a mysterious phenomenon. Why not just have the critical codon in the GluR2 gene set to encode R in the first place rather than go through the process of editing? We still do not know why the process evolved. Perhaps RNA editing represents a snapshot of an evolutionary transition to the genome. Peter's group also found that transcripts encoding the kainate receptor subunits were also partially edited and this influenced the calcium permeability of the various subunit combinations (Köhler et al., 1993). In fact, many targets for RNA editing beyond those transcripts encoding ionotropic glutamate receptors were subsequently discovered by Peter's group and others, and the editing process is overall important for healthy physiological function (Hartner et al., 2004; Horsch et al., 2011; Rosenthal and Seeburg, 2012).

## The max plank years

The offer to Peter of a prestigious directorship to head up a new Department of Molecular Neurobiology at the Max Planck Institute for Medical Research in Heidelberg, in parallel with Bert Sakmann's Department in the same institute, was well deserved. Around the same time Peter was also offered an MPI directorship in Munich, but he declined. So he and his colleagues moved to the Heidelberg MPI in 1996. At that time, he was around 52 years old, and had already achieved an enormous scientific impact and permanently cemented his reputation. The painstaking genetic work that subsequently followed from this MPI period was inevitably slower than perhaps the classical Seeburg style would have preferred, and possibly his impatient temperament would have been more suited to pursuing more proteomics-type studies (see quote at start of article!). In later years Peter wrote the following passage, which probably reflected his position at the start of his MPI period: “*A molecular neuroscientist with a pessimistic bent might feel that his field is coming to a close, seeing that most molecules contributing to a neuron's well-being and functional states are now known to us. There will still be unexplored ion channels and transcription factors in our genome, but isn't it merely a matter of time before these will be dragged onto the experimental stage? His colleague with an optimistic outlook, exulting in the great possibilities that molecular tools put at our disposal, will see himself more at the dawn of molecular neuroscience. The realist takes his position somewhere between these two extremes, realizing that he faces major challenges if he desires to contribute meaningfully to neuroscience by continuing to explore the molecular terrain*” (Seeburg, 2008).

In the subsequent 20 years following 1996, Peter and his colleagues Rolf Sprengel and Mia Higuchi used the freedom of MPI funding to publish collectively well over another 120 (!) papers, and expand further into manipulating the expression of the receptor subunits in transgenic and knockout mice, making knock-in point mutant mouse lines to reveal the critical importance of amino acid residues or whole regions in AMPA and NMDA receptor subunits (e.g., Sprengel et al., 1998; Krestel et al., 2004), and elucidating further the enzymology and sequence requirements of the brain RNA editing system, and highlighting the importance of RNA editing in the brain and other organs and tissues (Herb et al., 1996; Melcher et al., 1996b; Köhr et al., 1998; Seeburg et al., 1998; Feldmeyer et al., 1999; Higuchi et al., 2000; Hartner et al., 2004; Horsch et al., 2011). The Seeburg and Sprengel teams also collaborated with the mouse behavioral experts David Bannerman and Nick Rawlins at the University of Oxford, exploring the importance of hippocampal LTP for memory using, for example, mice with the NMDA receptor GluN1 gene deleted from dentate granule cells and CA1 pyramidal neurons (Bannerman et al., 2012, 2014). They found that although these hippocampal *Grin1* knockout mice had no LTP onto pyramidal cells or dentate granule cells they could still perform certain memory tasks, challenging the presumption that the NMDA receptor is essential for cognitive memory formation (Bannerman et al., 2012, 2014). Peter also continued to collaborate with former group member Hannah Monyer, who subsequently became his deepest friend and “Lebensmench,” and who looked after and cared for him during his long illness. Their last paper together, with joint PhD student Frauke Leitner, concerned studying the circuitry of olfactory processing in the entorhinal cortex (Leitner et al., 2016).

There was also some personal turbulence for Peter in the late 1990s with Genentech, with a legal dispute and trial centered around the ownership of patents and receipt of profits over recombinant growth hormone (Seeburg, 1999). Some colleagues, newspapers and magazines misunderstood or condemned Peter's position, invoking the deliberate misidentification of a plasmid for commercial reasons in a *Nature* paper. But not all was bleak: Peter was supported publically by Nüsslein-Vollhard and the science and its importance was never in dispute. As Peter wrote in a correspondence to *Nature*: “*As I emphasized during the trial, all scientific results and conclusions of the Nature paper (Goeddel et al.*, *1979**) are unambiguous and correct. The expression vector is exactly as described in the publication. The study in the paper forms the basis for the first human growth hormone preparation free of neurodegenerative agents, and the first recombinant therapeutic to be marketed by Genentech, from which 100,000 children benefit worldwide*” (Seeburg, 1999).

## Coda

Finally, let us finish by ascending Mount Olympus: Peter's metrics are impressive. Even those who dispute the value of metrics would have to concede that they do, in Peter's case, accurately describe the massive impact his body of work has had: an h-index of 140; 16 papers cited over a thousand times each, 24 papers cited above 500 times each, and a career total of about 84,000 citations and at least 342 publications (Sources: Web of Science and PubMed). He was elected an EMBO member in 1987. Peter garnered many prizes: the NEN Dupont Prize (1992), the Ernst Shering prize (1992), the TiPS/TINS lecture at the 1992 European Neuroscience Association (Seeburg, 1993), the Beckurts Prize (1992), the Feldberg prize (1993), a Bristol-Myers Squibb Unrestricted Award in Neuroscience (1997–1999), the 1997 ECNP-Lilly award, and in 2007 the InBev-Baillet Latour Health Prize. But Peter also gave to the community. He served on the edtrorial boards of numerous journals and of course, had contributed to the start, as its Chief Editor, of the *Frontiers in Molecular Neuroscience* journal where this article is published. Coming full circle, he served generously on the advisory board of the Chica and Heinz Schaller Foundation, an organization set up by his PhD supervisor, Heinz Schaller and Heinz's wife the developmental biologist Chica Schaller, to promote young career scientists and initiatives in neuroscience and infectious diseases.

In the early 1990s in Heidelberg, American Armed Forces radio was often on in the lab; when he heard a Jimi Hendrix track, Peter would come out of his office, turn up the lab radio, and play air guitar. The lab would stop and marvel. Hendrix was his all-time favorite, Peter had a picture of Hendrix on his office shelf, and for the postdocs and students in the lab, the rock musician seemed to sum up the scientist. Peter's metrics are a series of Golden Number 1's in the publication stakes. He had so many hits. Peter was a strict boss, but also a loving and loyal friend to some. He was often great fun and a shrewd observer. He enjoyed dining out, and had many entertaining anecdotes relating to science or personalities. He had a sharp wit and wicked chuckle. He could be undoubtedly difficult, but also deeply warm and generous. For the many students and postdocs in his group over the years, as their supervisor and mentor, he inspired them to achieve their best. Fittingly, his funeral service played out with Voodoo Child.

In Memory of Peter H Seeburg (21st August 1944–22nd August 2016) (Figure 1).

## Author contributions

The author confirms being the sole contributor of this work and approved it for publication.

## Funding

WW holds a joint Wellcome Trust Investigator award (together with N.P. Franks); 107841/Z/15/Z.

### Conflict of interest statement

The author declares that the research was conducted in the absence of any commercial or financial relationships that could be construed as a potential conflict of interest.
